# Perch time allocation and feeding efficiency of flycatching *Rhinolophus formosae*: an optimal foraging behavior?

**DOI:** 10.1186/s40850-021-00077-7

**Published:** 2021-05-08

**Authors:** Ya-Fu Lee, Yen-Min Kuo, Wen-Chen Chu, Yu-Hsiu Lin, Hsiang-Yi Chang, Hsing-Yi Chang, Wei-Ming Chen

**Affiliations:** 1 Department of Life Sciences, National Cheng Kung University, Tainan, 701 Taiwan; 2 Present addresses: Taiwan Endemic Species Research Institute, Nantou, Taiwan

**Keywords:** Flycatching, Foraging, Forests, Giving-up time, Horseshoe bats, Perch

## Abstract

**Background:**

Flycatching bats are species-rare and comprise predominantly horseshoe bats (Rhinolophidae). Their hang-and-wait foraging mode and long constant-frequency echolocation calls offer advantages in energetics and prey detection, and may enable them apt to foraging optimally, yet not much is known about the foraging behavior of flycatching bats. Thus we assessed the perch use and foraging performance in the field by one of the largest horseshoe bats, *Rhinolophus formosae*, and offered insights on their perch time allocation.

**Results:**

The perching-foraging behaviors of the bats did not differ significantly between forest settings, but the residence and giving-up time, mean attack, and attack rate were higher in the late spring-early summer, whereas the mean capture, capture rate, and attack efficiency were lower in the late summer when volant juveniles joined the nocturnal activity. The bats maintained flycatching and exhibited largely similar attack rates through the night with peak residence time around the midnight, but the capture rate and attack efficiency both reduced toward midnight and then increased toward the hours right before dawn. The attack rate was negatively correlated to the number of perches used and perch switch; by contrast, the capture rate was positively correlated with both factors. The total residence time at a site increased but mean residence time per perch decreased as the number of perches used and perch-switch increased. The giving-up time was inversely correlated to the attack rate and attack efficiency, and decreased with an increasing capture rate.

**Conclusions:**

The bats increased perch switch at lower attack rates in early spring, but switched less frequently in late spring and prime summer months when insect abundance is higher. By scanning through a broad angular range for prey detection, and switching more frequently among perches, *R. formosae* foraged with an increased capture rate, and were able to remain at the site longer by slightly reducing their mean residence time per perch. Our results concur with the predictions of optimal foraging theory for patch selection and offer implications for further exploration of the foraging behavior of flycatching horseshoe bats.

## Background

Foraging behavior has long been one of the central themes in examining the functional evolutionary ecology of bats [1–3], because of their diverse food habits associated with characteristic eco-morphological features and sensory systems [4, 5]. The question whether bats forage in accordance to or approximating the predictions of optimal foraging theory also arise naturally, where maximizing a net energy intake is generally assumed [6, 7]. Among animal-eating bats that account for over 70% of the entire order and comprise predominantly insect-eating species [8], particular attention has been paid to those that adopt the most common open and edge space aerial hawking tactic and then substrate gleaning tactic [6, 7, 9]. Most aerial foragers generally, if not entirely, may employ a strategy combining both selective and opportunistic modes, depending on the habitats and prey availability [10]. While broad diets containing diverse prey items appear in both aerial hawking and gleaning bats, more specialized and narrow diets are usually found in gleaning foragers (reviewed in [11, 12]), where such gleaning may occur from the ground or an object (i.e., narrow space active- or passive-gleaning), or from a water surface (i.e., edge trawling, e.g., *Myotis daubentonii*, [13]) [14].

Perch hunting is relatively uncommon in bats, occurring in only about two dozen or so species that are usually medium to large in size [5]. Perch hunting bats are typified by broad wingtips and lower aspect ratios that allow for maneuverable flight in more cluttered space with dense vegetation [15]. While some of them are narrow-space gleaning foragers [14], nearly two third of perch hunting species use a true flycatcher style, although mostly retain the use of aerial hunting by continuous flight [16], and some are capable of gleaning (e.g., *Rhinolophus blasii* [17]). Among those species which do adopt flycatching, horseshoe bats (*Rhinolophus* spp., Rhinolophidae) account for a predominant proportion. Their foraging has stronger associations with finer habitat features and vegetation structure, so is potentially more susceptible to habitat alteration and disturbance [16].

Compared to aerial hunting, perch hunting permits bats the possibility of a broader search angle (e.g., *R. rouxi*, [18]), a longer time duration between detecting and approaching prey [19], a more efficient energetics (e.g., *R. mehelyi*) [20], and an improved signal-to-noise ratio due to lower wind noise [21]. The typical long constant-frequency (CF) component of high duty cycle calls by horseshoe bats enable them to evaluate potential prey more effectively. For instance, horseshoe bats can discriminate insect wingbeats and classify insects by echolocation information alone [22, 23]. These advantages may contribute to the selectivity of insect prey in a more economic manner than most aerial searching foragers [24–26]. Thus, the foraging characteristics of perch hunting horseshoe bats may more likely conform to the predictions of optimal foraging behavior [7]. Perch hunting resembles a sit-and-wait foraging mode and is less mobile than aerial hawking, thus it is particularly intriguing to consider the effect of foragers’ selectivity on the time spent at a feeding perch and the timing of the decision to move to a new perch (i.e., the giving-up time [27, 28]). Yet, the foraging behavior of most flycatching horseshoe bats, particularly in field conditions, has attracted relatively little attention thus far [16].

Accordingly, the present study commenced by examining the perching and foraging behavior of flycatching *R. formosae*, known as the Formosan woolly horseshoe bat, in Eastern Asian tropical monsoon forests and exploring the changes in this behavior over space and time. It is an endemic species to Taiwan and one of the most restrictedly distributed among all rhinolophids [29]. Among all known flycatching horseshoe bats, *R. formosae* is one of the largest, but emits echolocation calls of the lowest frequency for its size [16] (except *R. paradoxolophus* [14]). Notably, it prefers edges and open forests to forest interiors [16], where forest edges and open forests generally represent background cluttered spaces, and forest interiors correspond to highly cluttered spaces [30]. Moreover, *R. formosae* relies almost exclusively on flycatching for foraging [31], and in forest interiors selectively uses perches associated with more open space, in which the features and vegetation structures are similar to those in edge/open forest sites [16].

With this regard, we set out to explore whether the foraging behavior of *R. formosae* differs between different forest settings, and predicted that the foraging performance (e.g., the attack rate, capture rate, and attack efficiency) would be higher in edge-open forest sites than in forest interiors. We then examined the perch use, perch time allocation, and hunting efficiency of *R. formosae* in order to test whether its foraging behavior conforms to the patch use model derived from optimal foraging theory [32]. In particular, we predicted that the bats would spend more time at sites of higher captures and would show an inverse giving-up time with increasing average capture rates [27].

## Results

A total of 463 foraging bouts of *R. formosae* were recorded through approximately 587.2 h of acoustic tracking-monitoring over 103 nights. On average, each bout lasted for 76.2 ± 3.37 min per bat-night (range: 1 ~ 665 min, *n* = 463). Overall, the perching and hunting behavior variables showed different degrees of variability in the edge-open forest sites and forest interior sites, respectively (Table 1).

**Table 1:** Mean (± SE) values of measured perching and foraging parameters for *R. formosae* in edge-open forest and forest interior sites in the GEF-HTBG forests, Kenting, Taiwan

Variables	Edge-open (*n* = 257 bouts)	Interior (*n* = 200 bouts)
**Perching variables**		
Perch	2.49 ± 0.13 (79.8; 253)	2.23 ± 0.13 (80.2; 195)
Perch-switch	2.55 ± 0.32 (198.5; 253)	1.99 ± 0.23 (159.7; 195)
Perch-switch rate	0.07 ± 0.01 (146.8; 251)	0.06 ± 0.01 (187.0; 194)
**Foraging variables**		
Site residence time	40.43 ± 3.18 (124.8; 252)	46.63 ± 3.67 (110.5; 195)
Perch residence time	12.97 ± 0.82 (99.8; 252)	17.77 ± 1.74 (137.7; 195)
Giving-up time	3.22 ± 0.23 (112.3; 242)	3.83 ± 0.64 (223.3; 178)
Site attack	13.60 ± 1.33 (155.2; 251)	17.2 ± 1.64 (133.7; 196)
Perch attack	4.10 ± 0.35 (113.5; 181)	5.63 ± 0.71 (143.3; 131)
Attack rate	0.28 ± 0.02 (95.1; 251)	0.31 ± 0.02 (86.8; 196)
Site capture	2.12 ± 0.73 (415.9; 147)	3.02 ± 0.51 (175.5; 106)
Perch capture	0.73 ± 0.15 (215.4; 110)	1.45 ± 0.32 (188.9; 75)
Capture rate	0.05 ± 0.01 (220.0; 145)	0.10 ± 0.02 (174.6; 99)
Attack efficiency	0.11 ± 0.02 (186.3; 145)	0.20 ± 0.03 (135.0; 98)

Coefficients of variation (%) and sample sizes (*n*) are in parentheses

### Perching and inter-perch movements

The observed bats almost always perched solitarily, unless carrying a baby (5 confirmed cases). On average, each bat used 2.4 ± 0.09 perches per site-night (range: 1 ~ 12; *n* = 450), and within each bout made 2.8 ± 0.22 perch-switch attempts (range: 0 ~ 63) and 2.3 ± 0.20 actual switches (range: 0 ~ 61; *n* = 450), giving at a perch-switch ratio of about 76.5%.

**Fig. 1:**
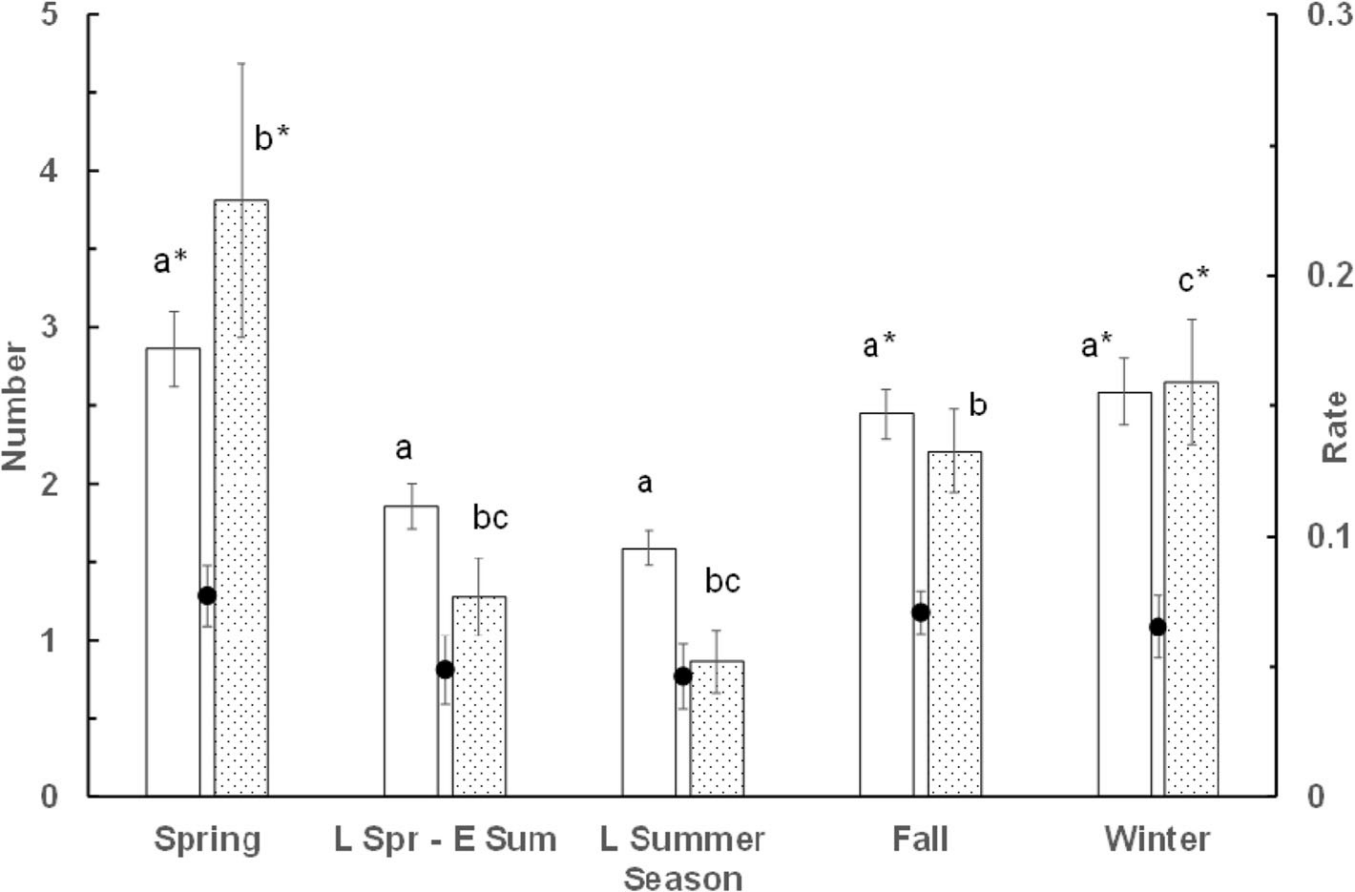
Mean (± *SE*) perch number (), perch switch (), and switch rate () per site by *Rhinolophus formosae* over different seasonal phases of the year in the GEF-HTBG forest, Kenting, Taiwan. A seasonal phase with a letter and an asterisk at a variable indicates a significantly higher value regarding that variable than other phases with the same letter but without an asterisk

The perching patterns of the bats varied with the season (MANOVA, Pillai-Bartlett’s *trace* value *V =* 0.051, *F*_12, 1281_ = 1.86, *p* < 0.05) but were not affected by the forest setting (edge-open or interior; *V =* 0.001, *F*_3, 425_ = 0.19, *p* > 0.5; factor × factor interaction: *V =* 0.028, *F*_12, 1281_ = 1.01, *p* > 0.5). The number of perch switches made by the bats peaked in early spring, then reduced over the summer months before rising once again in the fall and winter (Fig. 1). Thus, the bats tended to use more perches in the early spring (2.9 ± 0.24, *n* = 75, Fisher**’**s LSD, *p* < 0.001), fall (2.5 ± 0.11, *n* = 147, *p* < 0.005), and winter (2.6 ± 0.22, *n* = 110, *p* < 0.005) than in the late spring-early summer (1.9 ± 0.14, *n* = 64) or late summer (1.6 ± 0.11, *n* = 54; Fig. 1).

### Perch hunting and efficiency

Overall, *R. formosae* spent a residence time of 15.1 ± 0.90 min (range: 1 s ~ 172.8 min) at each perch and 43.2 ± 2.41 min (range: 1 s ~ 415.8 min; *n* = 449) at each site (multiple perches) per night. While on a perch, the bats spun around frequently and emitted echolocation calls constantly. The spinning motion covered various angular ranges, often up to 270° or so and took about 2.7 ± 0.08 s (*n* = 15). On average, each attack sally flight took ca. 2.88 ± 0.06 s (*n* = 247 bat-bouts over 4618 sally trips), peaking in the prime summer (3.32 ± 0.17 s; *F*_4, 688_ = 8.20, *p* < 0.001) and falling to a minimum in the winter months (2.45 ± 0.10 s; all paired comparisons *p* values < 0.05).

**Fig. 2:**
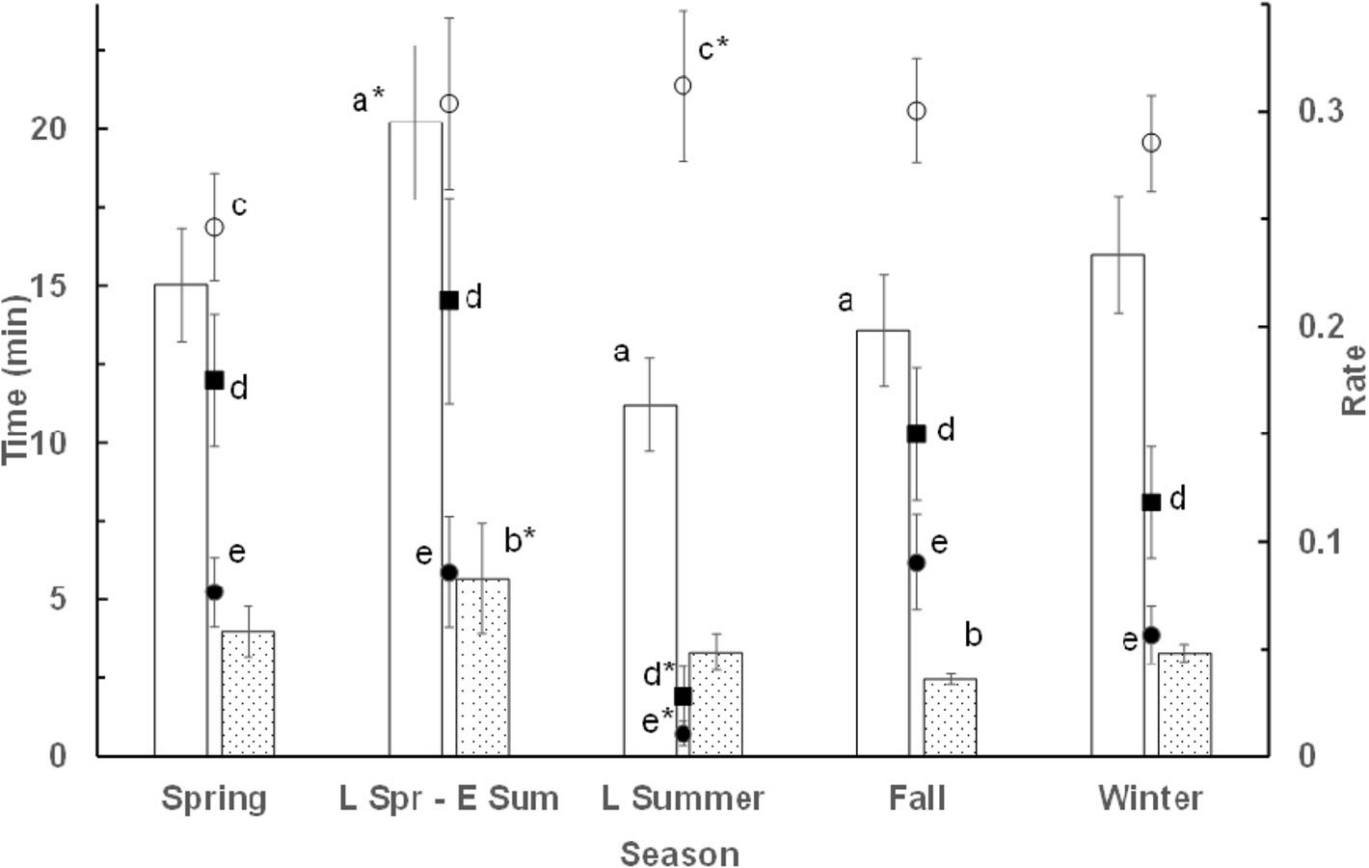
Mean (± *SE*) perch residence time (min, ), giving-up time (min, ), attack rate (#/min, ), capture rate (), and attack efficiency () of *R. formosae* over different periods of the year in the GEF-HTBG forest, Kenting, Taiwan. See Fig. 1 for the meaning of letters and asterisks in comparisons among seasonal phases for any variable

The perch hunting behavior of the bats also varied with the season (Pillai-Bartlett’s *trace* value *V =* 0.147, *F*_20, 880_ = 1.68, *p* < 0.05), but not with the forest setting (edge-open or interior; *V =* 0.025, *F*_5, 217_ = 1.09, *p* = 0.39; factor × factor interaction: *V =* 0.074, *F*_20, 880_ = 0.83, *p* = 0.68). The bats spent less time at a site, and showed a shorter mean perch residence time, in the late summer (11.2 ± 1.47 min, *n* = 54) and fall (13.6 ± 1.80 min, *n* = 146) than in the late spring-early summer (20.2 ± 2.46 min, *n* = 63; Fisher**’**s LSD, *p* < 0.05). By contrast, the giving-up time increased in the late spring-early summer (5.7 ± 1.75 min, *n* = 59), but fell to a minimum in the fall (2.5 ± 0.16 min, *n* = 138; Fisher**’**s LSD, *p* < 0.05; Fig. 2).

On average, the bats made 4.7 ± 0.36 (range: 0 ~ 49; *n* = 312) and 15.2 ± 1.04 (range: 0 ~ 229; *n* = 447) attack sallies per perch and per site, respectively, and exhibited an overall attack rate of 0.29 ± 0.01 (range: 0 ~ 2.42; *n* = 447) per min per perch-site. The lowest attack rate occurred in the spring (0.25 ± 0.03, *n* = 75; Fisher’s LSD, *p* < 0.05), while the peak attack rate occurred in the late summer (0.31 ± 0.04, *n* = 54; Fisher’s LSD, *p* < 0.05). Based on a conservative estimate, the bats achieved an overall capture rate of 0.07 ± 0.01 (range: 0 ~ 1.11; *n* = 244) per min at an attack efficiency of 15.5 ± 0.02% (*n* = 243). The highest attack rate was associated with the lowest mean capture rate (0.01 ± 0.006; Fisher’s LSD, *p*-values < 0.05 in all paired comparisons) and attack efficiency (0.03 ± 0.014; Fisher’s LSD, *p*-values < 0.05 in all paired comparison; Fig. 2).

### Perching time allocation and correlations

**Fig. 3:**
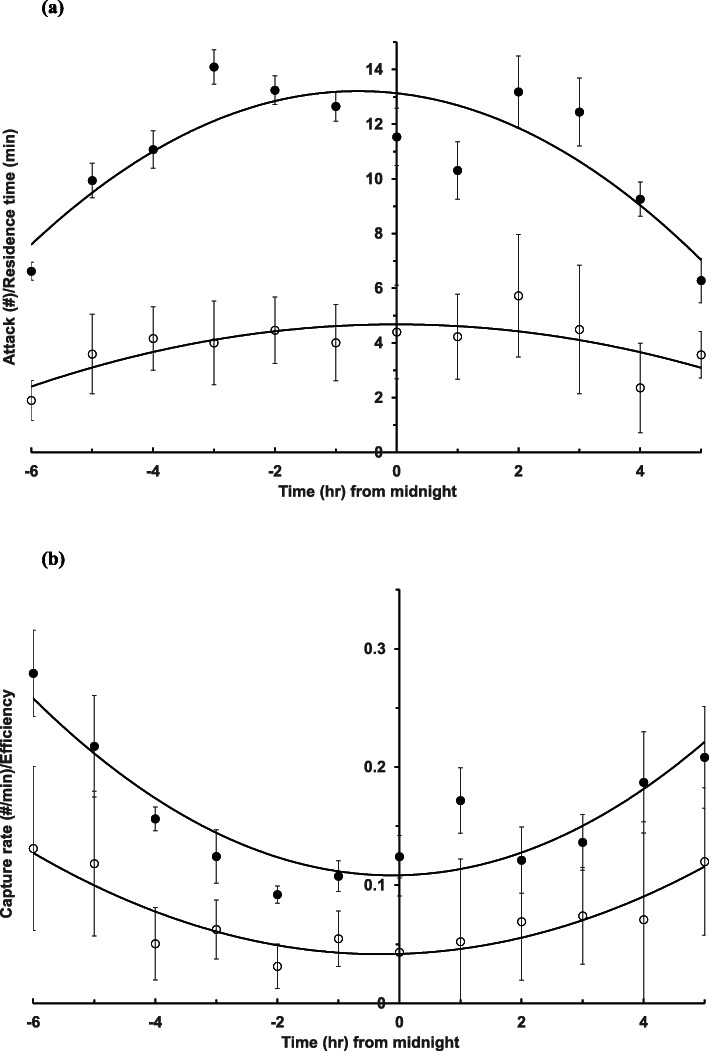
Mean (± *SE*) (**a**) attacks () and residence time () per perch, and (**b**) capture rate (; capture per min) and attack efficiency () of *R. formosae* over the night course in the GEF-HTBG forest, Kenting, Taiwan

Our evening sampling first located a bat perching on a tree about 4.9 ± 0.07 h (*n* = 55) before midnight, ranging from 4.07 h (June) to 6.08 h (December). Over the course of the night, the mean attacks of the bats varied convexly, with a peak occurring at around midnight (*R*^2^ = 0.73, *p* < 0.05). A similar tendency was noted for the residence time (*R*^2^ = 0.54, *p* < 0.05; Fig. 3a), resulting an approximately constant mean attack rate over time (*R*^2^ = 0.14). By contrast, the capture rate (*R*^2^ = 0.81, *p* < 0.05) and attack efficiency (*R*^2^ = 0.81, *p* < 0.05) both varied concavely over the night period (Fig. 3b).

**Fig. 4:**
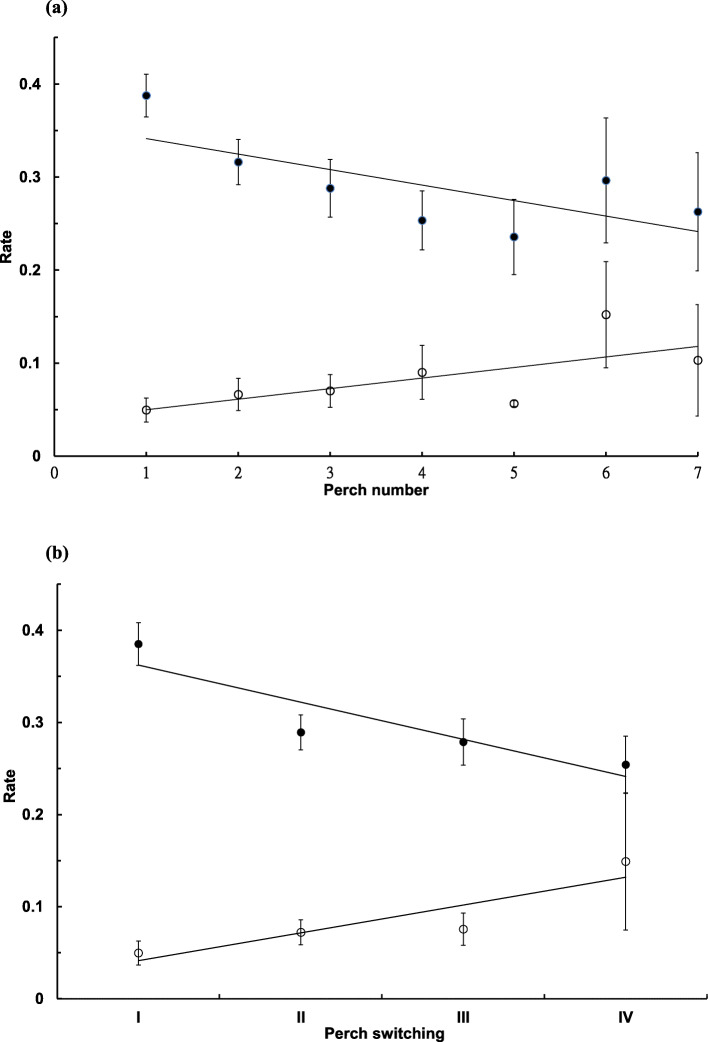
Mean (± *SE*) attack rate () and capture rate () of *R. formosae* in the GEF-HTBG forest, Kenting, Taiwan, for different (**a**) numbers of perches (7 or more perches were combined), and (**b**) levels of perch switching (I: 0; II: 1 ~ 3; III: 4 ~ 9; IV: > 10 times)

**Fig. 5:**
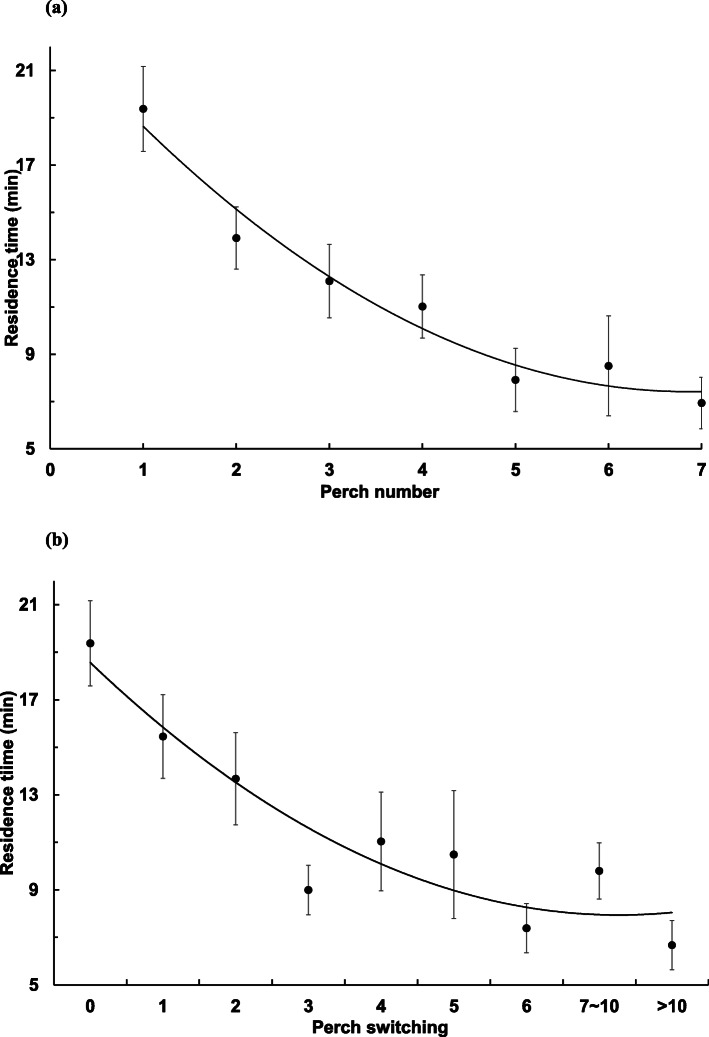
Mean (± *SE*) residence time of *R. formosae* in the GEF-HTBG forest, Kenting, Taiwan, for different numbers of (**a**) perch points and (**b**) perch switches

**Fig. 6:**
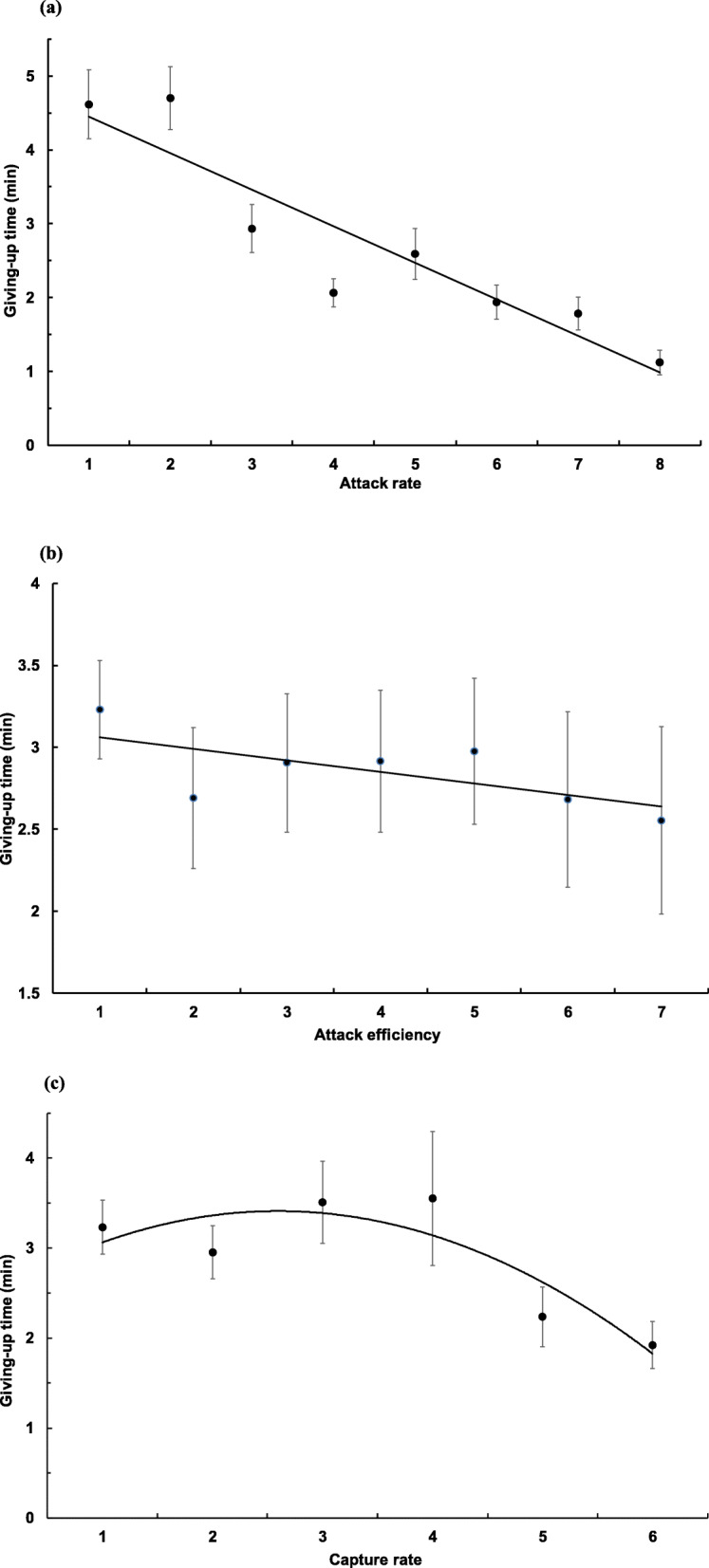
Mean (± *SE*) giving-up time of *R. formosae* in the GEF-HTBG forest, Kenting, Taiwan, for different levels of (**a**) attack rate (1: ≤ 0.1, 2: ≤ 0.2, 3: ≤ 0.3, 4: ≤ 0.4, 5: ≤ 0.5, 6: ≤ 0.6, 7: ≤ 0.7, 8: > 0.7), (**b**) attack efficiency (%; 1: 0, 2: ≤ 0.1, 3: ≤ 0.2, 4: ≤ 0.3, 5: ≤ 0.4, 6: ≤ 0.5, 7: > 0.5), and (**c**) capture rate (1: 0, 2: ≤ 0.05, 3: ≤ 0.1, 4: ≤ 0.15, 5: ≤ 0.2, 6: > 0.2)

The attack rate of *R. formosae* was negatively correlated, while the capture rate was positively correlated, with the number of perches actually taken (attack rate, *r* = − 0.71, *p* < 0.05; capture rate, *r* = 0.70, *p* < 0.05; Fig. 4a) and the level of perch switching (attack rate, *r* = − 0.91, *p* < 0.05; capture rate, *r* = 0.91, *p* < 0.05; Fig. 4b). The total residence time at a site increased with the number of perches used (*R*^2^ = 0.96) and the extent of perch switching (*R*^2^ = 0.82). By contrast, the mean residence time per perch declined curvilinearly with the number of perches used (*R*^2^ = 0.96, Fig. 5a), and the perch switching (*R*^2^ = 0.87, Fig. 5b). Finally, the giving-up time was negatively correlated with the attack rate (*r* = − 0.92, *p* < 0.05; Fig. 6a) and attack efficiency (*r* = − 0.67, *p* < 0.05; Fig. 6b), and decreased curvilinearly with the capture rate (*R*^2^ = 0.77, *F*_2, 8_ = 316.61, *p* < 0.001; Fig. 6c).

## Discussion

Previous studies have investigated the activity patterns, habitat use, food habits, and echolocation behavior of some flycatching bats (reviewed in [16]), particularly in Eurasian greater horseshoe bats, *R. ferrumenquinum* [24, 33–35]. The present study provides by far the most detailed insights yet on the perch use, perch time allocation, and foraging efficiency by flycatching horseshoe bats in the field. *Rhinolophus formosae* in the study area showed largely similar perching-foraging behaviors with no significant differences between the edge-open forests and forest interiors. This finding doesn’t supports our first prediction regarding the perching-foraging of the bats in the two forest settings. This result, however, is consistent with a previous finding that *R. formosae* in forest interiors selectively use perches associated with a certain extent of open space presumably permitting more sufficient prey detection and maneuverable sallies for prey capture [16].

Unlike the forest setting, the bats showed seasonal variation in perching and foraging behaviors. This finding results likely from a fluctuating prey abundance as well as, at least in part, the changes in the individual states of the bat composition over seasons. Insect abundance shows seasonality, even in the tropics [36], which is typically higher in the late spring to summer periods, but is less stable in early spring and the fall when ill-timed weather patterns may occur, and reduces to a minimum in the winter. Winter months coincide with the dry season in Kenting, and provides a harsh environment to many plants and insects. Thus, reproductive females, particularly those in the late pregnancy or lactation stage may spend less time switching among perches in order to reduce the travel cost and conserve their energy [37, 38]. Newly volant and sub-adult bats join the nocturnal perching and foraging activity in the late summer and fall. These bats are poorer in maneuverability and flight ability than adults due to their less well developed wing structure and flight muscles [39, 40]. Furthermore, they are less mature and stable in prey detection and discrimination [41]. As a result, their overall foraging performance is presumably poorer.

*Rhinolophus formosae* resembles *R. ferrumequinum* in body size, but is slightly larger in craniodental measurements (i.e., the mandible length, mastoid width, and upper and lower toothrow lengths [29]). They feed on similar major prey types (beetles and moths [11, 42, 43]), with the prey typically sized ca. 10 ~ 45 mm [24, 40]. The capture rate and attack efficiency observed in the present study may be underestimated and biased toward larger prey that would more likely be brought back to a perch, since small prey would be easily and quickly processed without being noticed, or even before the bats returning to the perch. In general, the proportion of large-sized insects tends to decline as moisture level increases in forests [44]. Thus, it is reasonable to expect a higher foraging activity and capture rate in edge-open forests than in forest interiors. In our study, however, the foraging variables did not differ between the two settings. This may likely result from the observations that although insect abundance increased in edge-open forest settings, the relative abundances of moths and beetles, the major preys of *R. formosae*, were higher in forest interior sites over most seasonal periods (YF Lee, unpubl. data).

The foraging of *R. ferrumequinum* begins by aerial hawking within a couple of hours after sunset [39], and is followed by a resting period [41, 45] before by flycatching with shorter foraging bouts between midnight and sunrise [45], which is similar to that of *R. rouxi* [18]. Like most other flycatching species e.g., *R. blasii* [17], *R. megaphyllus* [46], *R. mehelyi*, [47], *R. ferrumequinum* also hunts in open areas [48]. In contrast, *R. formosae* forages almost continuously, almost exclusively by flycatching, and seldom over open areas [31]. Their smaller aspect ratio, compared to other flycatching horseshoe bats such as *R. ferrumequinum* and *R. mehelyi*, may explain why the nocturnal activity of *R. formosae* appears more evenly distributed over time and space from a perspective of economic efficiency [16, 47]. Over the night course, however, *R. formosae* showed noticeable temporal variations, with the highest residence time but lowest attack efficiency and capture rate occurring around midnight when prey activity is generally low [49, 50]. This also concurs with the general bimodal foraging activity displayed by numerous aerial hawking bats [51].

*Rhinolophus formosae* resembles the smaller-sized *R. rouxi* in general perch use [16]. The bats, however, made longer attack sallies (ca. 2.9 s) than *R. rouxi* (ca. 1 s [18]), with a peak sally time in the prime summer months and a minimum sally time in the dry winter months. The longer sally flights of *R. formosae* suggest a longer range of prey detection, which is consistent with their exceptionally low CF-call frequency and frequent use of less cluttered edge-open forests for prey detection [16]. The constant strong wind in the winter season in Kenting [52] presumably interferes with the prey detection of bats by echolocation. This helps to explain the greater capture variability observed in the edge-open sites.

On average, *R. formosae* attacked at a slightly lower rate than *R. rouxi* (ca. 0.5 sally per min [18]); yet, the fastest sally rate (2.42 per min) by *R. formosae* was almost comparable to that of *R. rouxi* (4 sallies in 2 min). In addition, *R. formosae* resided longer (mean: 15.1 min, max: 172.8 min) and exhibited a broader spinning angle than *R. rouxi* (mean: 7 min, max: 34 min, spinning angle: ca. 200° [18]). It’s been speculated that the foraging mode is governed by prey abundance, suggesting the more energy-efficient perch hunting is the consequence of lower prey abundance [18]. Even long-term insect assessment at a broad spatial scale has yet to be performed in the study area, there is no evidence to suspect that prey abundance is exceptionally low. In contrast, the presence of a few species of edge and narrow space bats constantly foraging in the study area [31, 53] suggests a sufficient insect abundance.

By scanning over a broad angular range for prey detection and switching among perches frequently, our data indicate that *R. formosae* foraged with a reduced attack rate but increased capture rate as perch number and switch increased, and were able to remain longer at these multiple-perch sites (site residence time) by only slightly reducing the mean residence time per perch. This suggests an adaptive value of perch switch for flycatching bats, in addition to contributing to assessing the environment and information gathering. The giving-up times of the bats did not differ between the edge-open forest and forest interior sites, but were inversely correlated to the attack rate, attack efficiency, and capture rate. These results appear to concur with the predictions regarding the optimal patch choice for feeding (i.e., [54]) based on the marginal value theorem [32], namely the giving up time is shorter in better habitats where the average capture rate is higher [27, 28, 55].

The giving-up time, however, may not be the only or the best “moving-on threshold” or rule [54, 56], and a certain assessment of factors beyond energy may be involved. That *R. formosae* preferred perches of specific features and in forest interiors selectively used perches associated with more open space [16] indicates the bats do not forage at random. That the giving-up time negatively correlated with attack rate at a steeper slope than they were with capture rate and attack efficiency also suggests that the bats probably rely more on their own detection than on the outcome of an attack for evaluating the overall quality of sites. Failed attacks caused reduced capture rate and attack efficiency, but may still offer information regarding the prey availability. This concurs with the much smaller variability observed in giving-up time when plotted with attack rate than that with attack efficiency and capture rate.

Whether bats forage in a manner approximating to the predictions of optimal foraging theory has long been an intriguing question but remains controversial, mainly because of the constraints imposed on the time spent for detection and discrimination by virtue of the fact that both bats and potential preys are moving [57]. Within their foraging habitats, many aerial hawking bats appear to be flexible and opportunistic, pursuing the most abundant prey [10]. Recent modelling work with empirical data indicate that *Pipistrellus abramus* control sensing and navigation dynamically by distributing their attention among multiple preys, suggesting that bats may be capable of spatially anticipating future targets rather than simply foraging in a hit-or-miss fashion [58]. Intuitively, such a tactic is likely to increase the capture success and offer distinct advantages for typical opportunistic foraging. However, *M. daubentonii* was found selectively feeding on chironomid midges even in the presence of a significant increase in black fly availability [59], which suggests no support for optimally foraging on the most abundant preys. From the perspective of economic efficiency, neither opportunistic nor selective feeding may conflict with optimal foraging, depending on the cost-benefit ratio [5].

In contrast, flycatching or perch hunting resembles a sit-and-wait foraging mode [60]. Foragers adopting such a mode rely largely on moving prey. For this tactic to pay off, the prey should be relatively mobile, present in a sufficiently high density, or both, or the energy needs of the predator must be low [61]. Perch hunting is more economically efficient in energy use than continuous flight [20], thus concurs with a lower energy requirement. Unlike most other echolocating bats, the typical high duty cycle CF calls of horseshoe bats enable them to evaluate prey more effectively [22, 23]. That *R. ferrumequinu* is capable of adjusting their selectivity toward more profitable prey in accordance with their inter-arrival intervals has led to the prediction that horseshoe bats would be more selective when perch-hunting [26]. This may contribute in turn to their ability to adjust selectivity toward larger insect prey more effectively than actively searching foragers [24–26], and the perch switch behavior observed in this study.

## Conclusions

The present findings indicate that *R. formosae* performed increased perch switch at lower attack rates in early spring, but less frequent perch switch in late spring and prime summer months when insect abundance is higher. Frequent switch among perches improved foraging performance, allowing for a longer residence time, with the giving-up time negatively correlated to the attack rate, attack efficiency, and capture rate. This behavior is consistent with a sit-and-wait foraging mode. Further studies may test if flycatching horseshoe bats that are capable of categorizing prey from perches over a longer distance, and in sites of higher relative difference in prey abundance among patches, are more selective than those that lack a similar ability or reside in in sites lacking this resolution. The frog eating *T*. *cirrhosis*, also a perch hunting bat, shows a selective response to patches of various frog choruses [62]. This discriminating ability, however, is limited by relative differences between the chorus sizes, suggesting that further exploration is required to better understand how predators perceive their environment and prey availability, and the effects of this on foraging of flycatching bats.

## Methods

### Study area

The field study took place in the Hengchun Tropical Botanical Garden and the adjacent Guijijaou Experimental Forest (referred to hereafter as the GEF-HTBG forest; 120°48′E, 20°58′N, ca. 450 ha in area and 200 ~ 300 m in elevation) in Kenting, Taiwan. The area is located at the southern tip of the Hengchun Peninsula, and contains the largest remaining and least-disturbed lowland tropical monsoon forest in Taiwan. It is also among the northernmost of all the species-rich limestone karsts in Southeast Asia [63]. Formosan wooly horseshoe bats are year-round residents, but are largely solitary and low in overall abundance in the reef-karst areas of Kenting [53].

The area is typified by mean monthly temperatures of around 28 °C in mid-summer and over 20 °C in the coldest months. An annual precipitation of 2200-2300 mm is concentrated around mid-April to October, with particularly heavy rainfall occurring during the East Asian plum rain and typhoon seasons from May to September (data of Guijijaou Weather Station, TFRI). The dominant woody plants over the area consist mainly of various species of ebony *Diospyros* spp*.,* figs *Ficus* spp*.*, autumn maple trees *Bischofia javanica*, Formosan nato trees *Palaquium formosanum*, Philippine drypetes *Drypetes littoralis*, and Taiwan aglaia *Aglaia formosana*. The forest edges adjacent to the botanical garden additionally comprise patches of various types of native or introduced plants, including Cycadaceae, Lauraceae, Mimosaceae, Moraceae, Palmae, together with various ferns, lianas and vines [52, 53].

### Bat and perch sampling

Monthly acoustic surveys were conducted for 4-6 nights each month over a period of 24 months to search for *R. formosae.* We sampled an area of ca. 120 ha containing open forests, forest edges, and forest interiors using a mapped grid system (200 m × 200 m [53]). Using the grid, three transect lines in forest interiors, two transects in forest edges, and two transects in open forest sites were set up (length: 1145.7 ± 93.8 m each; 8.02 km in total), based on terrain and accessibility [31]. The forest-edge and open-forest sites represented background cluttered spaces, and the forest interiors were regarded as highly cluttered spaces [30, 64].

We used Pettersson D230 bat detectors (Pettersson Elektronik AB, Uppsala, Sweden) that were set in the heterodyne mode and tuned to the specific frequency range of *R. formosae*’s CF calls, ca. 39-42 kHz, to search for bats. Upon detecting recognizable signals, we slowly and quietly approached the call source and searched for the perching bat with the assistance of the available natural light or night vision instruments, as required. To minimize disturbance, we used warm yellowish light only and visible light was not shed directly upon the perching bats unless absolutely necessary. We followed one-way directions to search each transect line once only each night, but alternated the walking direction on any transect line every other time. We also cross-confirmed among searching teams based on routes and time to eliminate repeated sampling.

### Perching and foraging behaviors

Once a bat was located on a perch, we carried out acoustical monitoring and observations of the bat behavior, which continued until the bat left the site, whereupon a search for a new bat commenced. Each foraging bout comprised the acoustic monitoring and observations of a single bat resident at a specific site consisting of multiple perch locations within an acoustically trackable area of roughly 25 ~ 30 m in radius. In each bout, observations or measurements were made of both the perching and the foraging variables. The perching variables included the number of perches used, the inter-perch switch movements, and the switch rate. The foraging variables included the residence time, the giving-up time, the number of attack sallies, the attack rate, the number of captures, the capture rate, and the attack efficiency (Table 2). It was not possible to record data blind because our study involved focal animals in the field.

**Table 2:** Definitions of measured perching and foraging parameters for *R. formosae* in the GEF-HTBG forest, Kenting, Taiwan

Variables	
**Perching variables**	
Perch	Mean number of perch used per site-night
Perch-switch	Bats taking off and switched to another perch
Perch-switch rate	Number of switch per min residence time at a site
**Foraging variables**	
Site residence time (min)	Stay duration at a site per night
Perch residence time (min)	Stay duration at a perch per night
Giving-up time (min)	Time between the last capture and the leave
Site attack	Number of attack sally per site-night
Perch attack	Number of attack sally per perch-night
Attack rate	Number of attack per min at a perch
Site capture	Number of capture per site-night
Perch capture	Number of capture success per perch-night
Capture rate	Number of captures per min residence time at a perch
Attack efficiency	Number of capture relative to total number of attack sally

Bats taking off from a perch emit acoustic signals of a downward frequency [65, 66]. Furthermore, attack sallies can be distinguished from perch-switch flights based on their typically shorter duration and the presence of a feeding buzz at the end of signals (e.g., *R. rouxi*, [65]; *R. ferrumequinum*, [67]; YF Lee unpubl. data). In the present study, the bats often returned to the same perch after an attack sally, but occasionally performed a perch-switch flight immediately after. The bats sometimes returned to the same perch after a perch-switch flight. Thus, an examination of the return behavior was performed to determine the ratio of actual number of perch switches to the total number of perch switch attempts.

The giving-up time was defined as the time interval between the last attack of the bat and its final leave from the perch [28]. The capture success was evaluated based on the observed prey handling and chewing behavior and/or the chewing sounds detected by observers positioned underneath the perch approximately 3 ~ 5 m from the tree. There was no guarantee that every capture event was properly observed or identified, and every chewing activity detected. Thus the calculated capture success was recognized as providing only a conservative estimate at best. The observations and measurements were nevertheless taken as the basis for estimating the corresponding capture rate and attack efficiency (Table 2).

### Data analysis

Unless otherwise noted, all of the data reported in this study are presented as the mean ± standard error (*SE*) or relative proportion (%). Furthermore, all of the statistical tests were performed using STATISTICA 12 (StatSoft, Tulsa, Oklahoma) for Windows XP with an alpha value of 0.05. Proportional data were arcsine transformed to meet the assumption of normality [68]. A prior factor analysis was conducted to characterize the perching-hunting variables into two factor groups comprising independent variables for further analyses. In addition, the coefficient of variability (%) was estimated for each measured perching and foraging variable between the edge-open forest sties and forest interior sites. We performed Multivariate analysis of variance (MANOVA) tests with Pillai-Bartlett’s *trace* values (*V*) using a sigma-restricted parameterization to examine whether the forest setting and seasonal phases affected the inter-perch movement and foraging behavior and efficiency of the bats. Where significant variation was detected, we used Fisher’s least significant difference test (LSD) for unequal sample sizes to locate differences [68]. The duration of sally flights among seasonal phases was additionally compared by ANOVA. The temporal patterns of attacks, capture rate, and attack efficiency over nocturnal time periods, and the relationships between residence times with perch number and perch switch, respectively, were examined with polynomial regression analyses. Finally, the relationships between the attack rate and capture rate with perch number and perch switch, respectively, and those between the giving up times and attack rate and attack efficiency, respectively, were examined by correlation analyses.

## Data Availability

The data for this study are available from the corresponding author on personal request.
